# A time-delay COVID-19 propagation model considering supply chain transmission and hierarchical quarantine rate

**DOI:** 10.1186/s13662-021-03342-8

**Published:** 2021-03-30

**Authors:** Fangfang Yang, Zizhen Zhang

**Affiliations:** School of Management Science and Engineering, Anhui University of Finance and Economics, Bengbu, China

**Keywords:** Delays, Supply chain transmission, Hierachical quarantine rate, Bifurcation, Stability, SEIQR COVID-19 virus propagation model

## Abstract

In this manuscript, we investigate a novel Susceptible–Exposed–Infected–Quarantined–Recovered (SEIQR) COVID-19 propagation model with two delays, and we also consider supply chain transmission and hierarchical quarantine rate in this model. Firstly, we analyze the existence of an equilibrium, including a virus-free equilibrium and a virus-existence equilibrium. Then local stability and the occurrence of Hopf bifurcation have been researched by thinking of time delay as the bifurcation parameter. Besides, we calculate direction and stability of the Hopf bifurcation. Finally, we carry out some numerical simulations to prove the validity of theoretical results.

## Introduction

In 2019, there was a severe epidemic situation caused by a new corona virus in Wuhan, Hubei Province, China. The World Health Organization named this virus 2019-nCoV (COVID-19). As of December 6, 2020, about 67,381,994 COVID-19 patients have been diagnosed worldwide [1]. COVID-19 may lead to serious respiratory distress syndrome, multi-organ failure, septic shock and blood clots, and so on [2, 3]. Aside from the health problems, a range of other problems have emerged [4]. Owing to the highly infectiousness and invisibility of COVID-19, it has an enormous impact on the economy, the environment, the academic world, and so on. According to the survey report of Frontiers, a Swiss press publication, due to the outbreak of the epidemic, one fifth of the researchers interviewed said that the work could not be carried out at all, and researchers in South American countries are the most affected, with more than a third of researchers from Argentina, Chile and Brazil saying their work is completely impossible [5]. Hence, it has important implications for preventing and controlling COVID-19.

Mathematical models are usually used to depict the propagation of a virus [6–12], and so many scholars have proposed a lot of mathematical models to study the spreading laws of the COVID-19 virus [13–17]. Although strengthening personal protection helps to resist the virus [18], it is still very important to understand the law of virus transmission. Dynamic system models are beneficial for understanding the spreading laws of virus, so, many dynamic system models for the COVID-19 virus have been established [19–21]. A SIR model, which provided a theoretical framework to investigate its spread within a community, was developed by Cooper et al., and then the time evolution of different populations and diverse significant parameters for the spread of the disease in various communities had been studied [22]. Latency is the most common feature of viruses; based on this idea, Piovella proposed a new SEIR model to study simple analytical expressions for the peak and asymptotic values and their characteristic times of the populations affected by the COVID-19 pandemic [23]. The quarantine strategy, which has been widely used in the prevention of various diseases [24], is considered to be one of the most effective virus prevention measures. So, Rafiq et al. established a SEIQR model to describe the propagation of COVID-19 by taking the quarantine strategy into account, and they researched not only the equilibrium points and the reproduction number, but also the local and global asymptotic stability of the equilibria [25]:

1 $$ \textstyle\begin{cases} \frac{dS(t)}{dt}=\lambda -(\beta _{1}I(t)+\beta _{2}E(t))S(t)-\mu S(t), \\ \frac{dE(t)}{dt}=(\beta _{1}I(t)+\beta _{2}E(t))S(t)-(q_{1}+\kappa + \alpha +\mu )E(t), \\ \frac{dI(t)}{dt}=\alpha E(t)-(r+\mu +d_{1})I(t), \\ \frac{dQ(t)}{dt}=q_{1}E(t)-(q+\mu +d_{2})Q(t), \\ \frac{dR(t)}{dt}=\kappa E(t)+rI(t)+qQ(t)-\mu R(t), \end{cases} $$

where $S(t)$, $E(t)$, $I(t)$, $Q(t)$, $R(t)$ express the number of susceptible individuals, exposed individuals, infected individuals, quarantine individuals and recovered individuals at time *t*, respectively. The meanings of the remaining parameters in system (1) can be found in [25].

In the process of model analysis in [25], Rafiq et al. thought only exposed individuals would be quarantined, but in fact, infected individuals are more likely to be quarantined. And, since the exposed individuals may have no obvious symptoms of infection, the quarantine rate of exposed individuals is less than that of infected individuals. In Shandong Port-Qingdao Port, there were two workers, whose work was loading and unloading for imported cold chain products, infected with COVID-19. On November 9, 2020, some workers of Hailian cold storage in Tianjin Binhai New Area were detected to be infected with COVID-19 because of contact with imported pig elbows. So, COVID-19 can spread not only by infectious individuals, but also supply chain transmission with the virus. Therefore, it is increasingly important to study the influence of supply chain transmission when we investigate the propagation laws of COVID-19. On the one hand, exposed individuals have carried virus, but they do not show signs of infection immediately, such as an asymptomatic patient. Some exposed individuals may take about 24 days to turn into infected individuals, so, there is a latency delay before changing to infected individuals. On the other hand, when people who have been infected by the COVID-19 virus, including exposed individuals, infected individuals and quarantine individuals, convert into recovered individuals, they need a long time for treatment by chemotherapy and restoring. Thus, it is impossible for a person to recover immediately, and there exists a time delay. Considering the above ideas, we develop a SEIQR novel COVID-19 model with two delays and a hierarchical quarantine rate:

2 $$ \textstyle\begin{cases} \frac{dS(t)}{dt}=\lambda -(\beta _{1}I(t)+\beta _{2}E(t)+\beta _{3})S(t)- \mu S(t), \\ \frac{dE(t)}{dt}=(\beta _{1}I(t)+\beta _{2}E(t)+\beta _{3})S(t)-(q_{1}+ \mu )E(t)-\alpha E(t-\tau _{1})-\kappa E(t-\tau _{2}), \\ \frac{dI(t)}{dt}=\alpha E(t-\tau _{1})-(\mu +d_{1}+q_{2})I(t)-rI(t- \tau _{2}), \\ \frac{dQ(t)}{dt}=q_{1}E(t)+q_{2}I(t)-(\mu +d_{2})Q(t)-qQ(t-\tau _{2}), \\ \frac{dR(t)}{dt}=\kappa E(t-\tau _{2})+rI(t-\tau _{2})+qQ(t-\tau _{2})- \mu R(t). \end{cases} $$

There are some hypotheses for the model:

1. *λ* is the recruitment rate of individuals, $\lambda \neq 0$;
2. *μ* is the natural death rate of individuals;
3. *κ* and *r* represent the recovery rate of exposed individuals, infected individuals and quarantine individuals due to immunity, respectively;
4. $d_{1}$ and $d_{2}$ represent the death rate of infected individuals, quarantine individuals, respectively;
5. $\beta _{1}$ and $\beta _{2}$ represent the contact rate of susceptible individuals with exposed individuals and infected individuals, respectively; $\beta _{3}$ represents the infectious rate of susceptible individuals due to supply chain transmission;
6. $q_{1}$ is the quarantine rate of exposed individuals; $q_{2}$ is the quarantine rate of infected individuals; $q_{1}$ should be smaller than $q_{2}$;
7. $\tau _{1}$ is the latency delay before virus outbreak; $\tau _{2}$ is the time delay to treatment before the exposed individuals, infected individuals and quarantine individuals come into recovered.

The rest of the paper is arranged as follows: In Sect. 2, the existence of a virus-free equilibrium and a virus-existence equilibrium are discussed. In Sect. 3, we take two delays as bifurcation parameters, and local stability of the virus-existence equilibrium and the occurrence of Hopf bifurcation are analyzed. In Sect. 4, the direction and stability of the Hopf bifurcation when $\tau _{1}>\tau _{2}$ and $\tau _{2}\in (0,\tau _{20})$ are examined, especially. In Sect. 5, we test the validity of the theoretical results. We summarize our work in Sect. 6.

## The existence of equilibrium

At first, the existence of a virus-free equilibrium is discussed. Assume that system (2) has a virus-free equilibrium $G_{0}(S_{0}, E_{0}, I_{0}, Q_{0}, R_{0})$, and $E_{0}=I_{0}=0$, $S_{0}\geq 0$, $Q_{0}\geq 0$, $R_{0}\geq 0$. So we can obtain the following equations:

3 $$ \textstyle\begin{cases} \lambda -(\beta _{1}I_{0}+\beta _{2}E_{0}+\beta _{3})S_{0}-\mu S_{0}=0, \\ (\beta _{1}I_{0}+\beta _{2}E_{0}+\beta _{3})S_{0}-(q_{1}+\mu )E_{0}- \alpha E_{0}-\kappa E_{0}=0, \\ \alpha E_{0}-(\mu +d_{1}+q_{2})I_{0}-rI_{0}=0, \\ q_{1}E_{0}+q_{2}I_{0}-(\mu +d_{2})Q_{0}-qQ_{0}=0, \\ \kappa E_{0}+rI_{0}+qQ_{0}-\mu R_{0}=0. \end{cases} $$

After calculation, we can get $S_{0}=0$ from the second equation. Taking $S_{0}=0$ in the first equation, we can obtain $\lambda =0$. In fact, *λ* is the recruitment rate of individuals. It means that $\lambda \neq 0$. So, it is inconsistent with the facts, and system (2) has no virus-free equilibrium.

Then we analyze the existence of the virus-existence equilibrium $G^{*}(S^{*}, E^{*}, I^{*}, Q^{*}, R^{*})$. Let us equate the equations in system (2) to be zero, we obtain

4 $$\begin{aligned}& \textstyle\begin{cases} \lambda -(\beta _{1}I(t)+\beta _{2}E(t)+\beta _{3})S(t)-\mu S(t)=0, \\ (\beta _{1}I(t)+\beta _{2}E(t)+\beta _{3})S(t)-(q_{1}+\mu )E(t)- \alpha E(t)-\kappa E(t)=0, \\ \alpha E(t)-(\mu +d_{1}+q_{2})I(t)-rI(t)=0, \\ q_{1}E(t)+q_{2}I(t)-(\mu +d_{2})Q(t)-qQ(t)=0, \\ \kappa E(t)+rI(t)+qQ(t)-\mu R(t)=0, \end{cases}\displaystyle \\& S^{*}=\frac{\lambda -k_{3}E^{*}}{\mu }, \\& E^{*}=\frac{\lambda \beta _{3}}{K}, \\& I^{*}=\frac{\alpha E^{*}}{k_{1}}, \\& Q^{*}=\frac{q_{1}E^{*}+q_{2}I^{*}}{k_{2}}, \\& R^{*}=\frac{1}{\mu }\bigl(\kappa E^{*}+rI^{*}+qQ^{*} \bigr), \end{aligned}$$

where

$$\begin{aligned}& k_{1}=\mu +d_{1}+q_{2}+r, \\& k_{2}=\mu +d_{2}+q, \\& k_{3}=q_{1}+\mu +\alpha +\kappa , \\& k_{4}=q+\mu , \\& K=k_{3}-\frac{\alpha \beta _{1}}{k_{1}}+\mu (q_{1}+\mu +\alpha + \kappa )-\beta _{2}. \end{aligned}$$

According to the above analysis, system (2) has a unique virus-existence equilibrium $G^{*}(S^{*}, E^{*}, I^{*}, Q^{*}, R^{*})$.

In other words, system (2) has no virus-free equilibrium, and has only a virus-existence equilibrium $G^{*}(S^{*}, E^{*}, I^{*}, Q^{*}, R^{*})$.

## Local stability of the virus-existence equilibrium and the occurrence of Hopf bifurcation

We know system (2) has a virus-existence equilibrium, and the linearized part of system (2) is

5 $$ \textstyle\begin{cases} \frac{dS(t)}{dt}=a_{11}S(t)+a_{12}E(t)+a_{13}I(t), \\ \frac{dE(t)}{dt}=a_{21}S(t)+a_{22}E(t)+b_{22}E(t-\tau _{1})+c_{22}E(t- \tau _{1})+a_{23}I(t), \\ \frac{dI(t)}{dt}=b_{32}E(t-\tau _{1})+a_{33}I(t)+c_{33}I(t-\tau _{2}), \\ \frac{dQ(t)}{dt}=a_{42}E(t)+a_{43}I(t)+a_{44}Q(t)+c_{44}Q(t-\tau _{2}), \\ \frac{dR(t)}{dt}=c_{52}E(t-\tau _{2})+c_{53}I(t-\tau _{2})+c_{54}Q(t- \tau _{2})+a_{55}R(t), \end{cases} $$

where

$$\begin{aligned}& a_{11}=-(\beta _{1}I+\beta _{2}E+\beta _{3})-\mu , \qquad a_{12}=-\beta _{2}S, a_{12}=-\beta _{1}S, \\& a_{21}=\beta _{1}I+\beta _{2}E+\beta _{3},\qquad a_{22}=-(q_{1}+ \mu ), \\& b_{22}=-\alpha ,\qquad c_{22}=-\kappa ,\qquad b_{32}=\alpha ,\qquad a_{33}=-(\mu +d_{1}+q_{2}), \\& c_{33}=-r,\qquad a_{42}=q_{1},\qquad a_{43}=q_{2},\qquad a_{44}=-(\mu +d_{2}), \\& c_{44}=-q,\qquad c_{52}=\kappa ,\qquad c_{53}=\gamma ,\qquad c_{54}=q,\qquad a_{55}=-\mu . \end{aligned}$$

From the system (4), we can obtain the Jacobian matrix at the virus-existence equilibrium $G^{*}(S^{*}, E^{*}, I^{*}, Q^{*}, R^{*})$:

$$J\left(G^{*}\right)=\left(\begin{array}{ccccc} a_{11} & a_{12} & a_{13} & 0 & 0\\ a_{21} & a_{22}+b_{22}e^{-\lambda \tau_{1}}+c_{22}e^{-\lambda \tau_{2}} & a_{23} & 0 & 0\\ 0 & b_{32}e^{-\lambda \tau_{1}} & a_{33}+c_{33}e^{-\lambda \tau_{2}} & 0 & 0\\ 0 & a_{42} & a_{43} & a_{44}+c_{44}e^{-\lambda \tau_{2}} & 0\\ 0 & c_{52}e^{-\lambda \tau_{2}} & c_{53}e^{-\lambda \tau_{2}} & c_{54}e^{-\lambda \tau_{2}} & a_{55} \end{array}\right).$$

Then the corresponding characteristic equation can be obtained:

6 $$\begin{aligned}& F_{0}(\lambda )+F_{1}(\lambda )e^{-\lambda \tau _{1}}+F_{2}( \lambda )e^{-\lambda \tau _{2}}+F_{3}( \lambda )e^{-\lambda (\tau _{1}+ \tau _{2})} \\& \quad {}+F_{4}(\lambda )e^{-2\lambda \tau _{2}}+F_{5}(\lambda )e^{-\lambda ( \tau _{1}+2\tau _{2})}+F_{6}(\lambda )e^{-3\lambda \tau _{2}}=0, \end{aligned}$$

where

$$\begin{aligned}& F_{0}(\lambda )=(\lambda -a_{11}) (\lambda -a_{22}) (\lambda -a_{33}) ( \lambda -a_{44}) (\lambda -a_{55}) \\& \hphantom{F_{0}(\lambda )=}{}-a_{12}a_{21}(\lambda -a_{33}) ( \lambda -a_{44}) (\lambda -a_{55}), \\& F_{1}(\lambda )=-b_{22}(\lambda -a_{11}) ( \lambda -a_{33}) (\lambda -a_{44}) ( \lambda -a_{55})-a_{23}b_{32}(\lambda -a_{11}) (\lambda -a_{44}) ( \lambda -a_{55}) \\& \hphantom{F_{1}(\lambda )=}{}-a_{13}a_{21}b_{32}( \lambda -a_{44}) (\lambda -a_{55}), \\& F_{2}(\lambda )=-c_{22}(\lambda -a_{11}) ( \lambda -a_{33}) (\lambda -a_{44}) ( \lambda -a_{55}) \\& \hphantom{F_{2}(\lambda )=}{}-c_{33}(\lambda -a_{11}) (\lambda -a_{22}) (\lambda -a_{44}) ( \lambda -a_{55}) \\& \hphantom{F_{2}(\lambda )=}{}-c_{44}(\lambda -a_{11}) (\lambda -a_{22}) (\lambda -a_{33}) (\lambda -a_{55})+a_{12}a_{21}c_{44}( \lambda -a_{33}) (\lambda -a_{55}) \\& \hphantom{F_{2}(\lambda )=}{}+a_{12}a_{21}c_{33}( \lambda -a_{44}) (\lambda -a_{55}), \\& F_{3}(\lambda )=b_{22}c_{33}(\lambda -a_{11}) (\lambda -a_{44}) ( \lambda -a_{55})+a_{13}a_{21}b_{32}c_{44}( \lambda -a_{55}) \\& \hphantom{F_{3}(\lambda )=}{}+a_{23}b_{32}c_{44}( \lambda -a_{11}) (\lambda -a_{55}) \\& \hphantom{F_{3}(\lambda )=}{}+b_{22}c_{44}(\lambda -a_{11}) (\lambda -a_{33}) (\lambda -a_{55}), \\& F_{4}(\lambda )=c_{22}c_{33}(\lambda -a_{11}) (\lambda -a_{44}) ( \lambda -a_{55})+c_{22}c_{44}( \lambda -a_{11}) (\lambda -a_{33}) ( \lambda -a_{55}) \\& \hphantom{F_{4}(\lambda )=}{}+c_{33}c_{44}(\lambda -a_{11}) (\lambda -a_{22}) (\lambda -a_{55})-a_{12}a_{21}c_{33}c_{44}( \lambda -a_{55}), \\& F_{5}(\lambda )=-b_{22}c_{33}c_{44}( \lambda -a_{11}) (\lambda -a_{55}), \\& F_{6}(\lambda )=-c_{22}c_{33}c_{44}( \lambda -a_{11}) (\lambda -a_{55}). \end{aligned}$$

*Case* 1. $\tau _{1}=\tau _{2}=0$, Eq. (6) becomes

7 $$\begin{aligned}& \lambda ^{5}+\bigl(F_{0}^{4}+F_{1}^{4}+F_{2}^{4} \bigr)\lambda ^{4}+\bigl(F_{0}^{3}+F_{1}^{3}+F_{2}^{3}+F_{3}^{3}+F_{4}^{3} \bigr) \lambda ^{3} \\& \quad {}+\bigl(F_{0}^{2}+F_{1}^{2}+F_{2}^{2}+F_{3}^{2}+F_{4}^{2}+F_{5}^{2}+F_{6}^{2} \bigr) \lambda ^{2} \\& \quad {}+\bigl(F_{0}^{1}+F_{1}^{1}+F_{2}^{1}+F_{3}^{1}+F_{4}^{1}+F_{5}^{1}+F_{6}^{1} \bigr) \lambda +\bigl(F_{0}^{0}+F_{1}^{0}+F_{2}^{0}+F_{3}^{0}+F_{4}^{0}+F_{5}^{0}+F_{6}^{0} \bigr)=0, \end{aligned}$$

where $F_{i}^{j}$ ($i=0, 1, 2, 3, 4, 5, 6$; $j=0, 1, 2, 3, 4$) represents the coefficient of $\lambda ^{j}$ in $F_{i}(\lambda )$.

### Lemma 1

*According to the Routh–Hurwitz criteria*, *when* $\tau _{1}=\tau _{2}=0$, *the virus*-*existence equilibrium* $G^{*}(S^{*}, E^{*}, I^{*}, Q^{*}, R^{*})$ *is locally asymptotically stable*.

*Case* 2. $\tau _{1}>0$, $\tau _{2}=0$. Then Eq. (6) becomes

8 $$ \bigl[F_{0}(\lambda )+F_{2}(\lambda )+F_{4}(\lambda )+F_{6}(\lambda )\bigr]+ \bigl[F_{1}( \lambda )+F_{3}(\lambda )+F_{5}(\lambda )\bigr]e^{-\lambda \tau _{1}}=0. $$

Let $\lambda =i\omega _{1}$. Separating the real and imaginary parts, we obtain

9 $$ \textstyle\begin{cases} M_{11}\cos \tau _{1}\omega _{1}+M_{12}\sin \tau _{1}\omega _{1}=-N_{11}, \\ M_{12}\cos \tau _{1}\omega _{1}-M_{11}\sin \tau _{1}\omega _{1}=-N_{12}, \end{cases} $$

with

$$\begin{aligned}& M_{11}=\bigl(F_{1}^{0}+F_{3}^{0}+F_{5}^{0} \bigr)-\bigl(F_{1}^{2}+F_{3}^{2}+F_{5}^{2} \bigr) \omega _{1}^{2}+F_{1}^{4} \omega _{1}^{4}, \\& M_{12}=\bigl(F_{1}^{1}+F_{3}^{1}+F_{5}^{1} \bigr)\omega _{1}-\bigl(F_{1}^{3}+F_{3}^{3} \bigr) \omega _{1}^{3}, \\& N_{11}=\bigl(F_{0}^{0}+F_{2}^{0}+F_{4}^{0}+F_{6}^{0} \bigr)-\bigl(F_{0}^{2}+F_{2}^{2}+F_{4}^{2}+F_{6}^{2} \bigr) \omega _{1}+\bigl(F_{0}^{4}+F_{2}^{4} \bigr)\omega _{1}, \\& N_{12}=\bigl(F_{0}^{0}+F_{2}^{0}+F_{4}^{0}+F_{6}^{0} \bigr)\omega _{1}-\bigl(F_{0}^{3}+F_{2}^{3}+F_{4}^{3} \bigr) \omega _{1}^{3}. \end{aligned}$$

Squaring both sides of two equations in Eq. (9), and summing them up, Eq. (10) can be obtained

10 $$\begin{aligned} M_{11}^{2}+M_{12}^{2}=N_{11}^{2}+N_{12}^{2}. \end{aligned}$$

We suppose that $(P_{0})$: Eq. (10) has at least one positive real root $\omega _{10}$. Solving Eq. (10), we obtain

11 $$ \tau _{1}^{(i)}=\frac{1}{\omega _{10}} \times \biggl[\arccos \frac{M_{11}N_{11}+M_{12}N_{12}}{M_{11}^{2}+M_{12}^{2}}+2i\pi \biggr],\quad i=0, 1, 2, \ldots . $$

For convenience, we define

12 $$ \tau _{10}=\min \bigl\{ \tau _{1}^{(i)}, i=0, 1, 2, \ldots \bigr\} , $$

where $\tau _{1}^{(i)}$ is defined by Eq. (11).

Taking the derivative to $\tau _{1}$ of *λ*, we can get

13 $$ \biggl[\frac{d\lambda }{d\tau _{1}} \biggr]^{-1}= \frac{[F_{0}^{\prime }(\lambda )+F_{2}^{\prime }(\lambda )+F_{4}^{\prime }(\lambda )+F_{6}^{\prime }(\lambda )]+[F_{1}^{\prime }(\lambda )+F_{3}^{\prime }(\lambda )+F_{5}^{\prime }(\lambda )]e^{-\lambda \tau _{1}}}{\lambda [F_{1}(\lambda )+F_{3}(\lambda )+F_{5}^{\prime }(\lambda )]e^{-\lambda \tau _{1}}}- \frac{\tau _{1}}{\lambda }. $$

According to [26], when the hypothesis $(P_{1})$: $\operatorname{Re}[d\lambda /d\tau _{1}]^{-1}_{\tau _{1}=\tau _{10}}\neq 0$ holds, the virus-existence equilibrium $G^{*}(S^{*}, E^{*}, I^{*}, Q^{*}, R^{*})$ is locally asymptotically stable. So, we have Theorem 1.

### Theorem 1

*For system* (2), *when the hypotheses* $(P_{0})$*–*$(P_{1})$ *hold true*, *then* $G^{*}(S^{*}, E^{*}, I^{*}, Q^{*}, R^{*})$ *is locally asymptotically stable when* $\tau _{1}\in [0, \tau _{10})$; *system* (2) *undergoes a Hopf bifurcation at* $G^{*}(S^{*}, E^{*}, I^{*}, Q^{*}, R^{*})$ *when* $\tau _{1}=\tau _{10}$, *once* $\tau _{1}$ *exceeds* $\tau _{10}$, *system* (2) *becomes unstable*.

*Case* 3. $\tau _{1}=0$, $\tau _{2}>0$. Then Eq. (6) becomes

14 $$ \bigl[F_{0}(\lambda )+F_{1}(\lambda ) \bigr]+\bigl[F_{2}(\lambda )+F_{3}(\lambda ) \bigr]e^{- \lambda \tau _{2}}+\bigl[F_{4}(\lambda )+F_{5}( \lambda )\bigr]e^{-2\lambda \tau _{2}}+F_{6}(\lambda )e^{-3\lambda \tau _{2}}=0. $$

Multiplying $e^{\lambda \tau _{2}}$ on both sides of Eq. (14), we obtain

15 $$ \bigl[F_{0}(\lambda )+F_{1}(\lambda ) \bigr]e^{\lambda \tau _{2}}+\bigl[F_{2}( \lambda )+F_{3}( \lambda )\bigr]+\bigl[F_{4}(\lambda )+F_{5}(\lambda ) \bigr]e^{- \lambda \tau _{2}}+F_{6}(\lambda )e^{-2\lambda \tau _{2}}=0. $$

Taking $\lambda =i\omega _{2}$ into Eq. (15), we obtain

16 $$ \textstyle\begin{cases} (M_{21}+M_{23})\cos \tau _{2}\omega _{2}+(M_{22}+M_{24})\sin \tau _{2} \omega _{2}+N_{21} \\ \quad =-M_{25}\cos 2\tau _{2}\omega _{2}-M_{26}\sin 2 \tau _{2}\omega _{2}, \\ (M_{24}-M_{22})\cos \tau _{2}\omega _{2}+(M_{21}-M_{23})\sin \tau _{2} \omega _{2}+N_{22} \\ \quad =-M_{26}\cos 2\tau _{2}\omega _{2}+M_{25}\sin 2 \tau _{2}\omega _{2}, \end{cases} $$

with

$$\begin{aligned}& M_{21}=\bigl(F_{0}^{0}+F_{1}^{0} \bigr)-\bigl(F_{0}^{2}+F_{1}^{2} \bigr)\omega _{2}^{2}+\bigl(F_{0}^{4}+F_{1}^{4} \bigr) \omega _{2}^{4}, \\& M_{22}=\bigl(F_{0}^{3}+F_{1}^{3} \bigr)\omega _{2}^{3}-\bigl(F_{0}^{1}+F_{1}^{1} \bigr) \omega _{2}-\omega _{2}^{5}, \\& M_{23}=\bigl(F_{4}^{0}+F_{5}^{0} \bigr)-\bigl(F_{4}^{2}+F_{5}^{2} \bigr)\omega _{2}^{2}, \\& M_{24}=\bigl(F_{4}^{1}+F_{5}^{1} \bigr)\omega _{2}-F_{4}^{3}\omega _{2}^{3}, \\& M_{25}=F_{6}^{0}-F_{6}^{2} \omega _{2}^{2}, \\& M_{26}=F_{6}^{1}\omega _{2}, \\& N_{21}=F_{2}^{0}+F_{3}^{0}- \bigl(F_{2}^{2}+F_{3}^{2} \bigr)\omega _{2}^{2}+F_{2}^{4} \omega _{2}^{4}, \\& N_{22}=\bigl(F_{2}^{1}+F_{3}^{1} \bigr)\omega _{2}-\bigl(F_{2}^{3}+F_{3}^{3} \bigr) \omega _{2}^{3}. \end{aligned}$$

Squaring both sides of two equations in Eq. (16), and adding them up, we obtain

17 $$\begin{aligned}& M_{21}^{2}+M_{22}^{2}+M_{23}^{2}+M_{24}^{2}-M_{25}^{2}-M_{26}^{2}+N_{21}^{2}+N_{22}^{2} \\& \quad {}+2(M_{21}M_{23}-M_{22}M_{24}) \bigl[2( \cos \omega _{2}\tau _{2})^{2}-1 \bigr] \\& \quad {}+2N_{21}(M_{22}+M_{24})\sin \omega _{2}\tau _{2}+2N_{22}(M_{21}-M_{23}) \sin \omega _{2}\tau _{2} \\& \quad {}+2N_{22}(M_{24}-M_{22})\cos \omega _{2}\tau _{2} \\& \quad {}+4(M_{22}M_{23}+M_{21}M_{24}) \cos \omega _{2}\tau _{2}\sin \omega _{2} \tau _{2}+2N_{21}(M_{21}+M_{23}) \cos \omega _{2}\tau _{2}=0. \end{aligned}$$

We have $\cos ^{2}\tau _{2}\omega _{2}+\sin ^{2}\tau _{2}\omega _{2}=1$, $\sin \tau _{2}\omega _{2}=\pm \sqrt{1-\cos ^{2}\tau _{2}\omega _{2}}$.

1. If $\sin \tau _{2}\omega _{2}=\sqrt{1-\cos ^{2}\tau _{2}\omega _{2}}$, after calculation, we have
  18 $$\begin{aligned}& M_{21}^{2}+M_{22}^{2}+M_{23}^{2}+M_{24}^{2}-M_{25}^{2}-M_{26}^{2}+N_{21}^{2}+N_{22}^{2} \\& \quad {}+2(M_{21}M_{23}-M_{22}M_{24}) \bigl[2( \cos \omega _{2}\tau _{2})^{2}-1 \bigr] \\& \quad {}+4(M_{22}M_{23}+M_{21}M_{24}) \cos \omega _{2}\tau _{2}\sqrt{1-\cos ^{2} \tau _{2}\omega _{2}}+2N_{21}(M_{21}+M_{23}) \cos \omega _{2}\tau _{2} \\& \quad {}+2N_{21}(M_{22}+M_{24})\sqrt{1-\cos ^{2}\tau _{2}\omega _{2}}+2N_{22}(M_{21}-M_{23}) \sqrt{1-\cos ^{2}\tau _{2}\omega _{2}} \\& \quad {}+2N_{22}(M_{24}-M_{22})\cos \omega _{2}\tau _{2}=0. \end{aligned}$$

Let $f_{1}(\omega _{2})=\cos \tau _{2}\omega _{2}$, and we suppose that $(P_{2})$: $f_{1}(\omega _{2})=\cos \tau _{2}\omega _{2}$ has at least a positive root $\omega _{21}$, which makes Eq. (18) hold. Thus,

19 $$ \tau _{21}^{(i)}=\frac{1}{\omega _{21}} \times \bigl[\arccos \bigl(f_{1}(\omega _{21})\bigr)+2i \pi \bigr], \quad i=0, 1, 2, \ldots . $$

(2) If $\sin \tau _{2}\omega _{2}=-\sqrt{1-\cos ^{2}\tau _{2}\omega _{2}}$, in the same way, we have
  20 $$\begin{aligned}& M_{21}^{2}+M_{22}^{2}+M_{23}^{2}+M_{24}^{2}-M_{25}^{2}-M_{26}^{2}+N_{21}^{2}+N_{22}^{2} \\& \quad {}+2(M_{21}M_{23}-M_{22}M_{24}) \bigl[2( \cos \omega _{2}\tau _{2})^{2}-1 \bigr] \\& \quad {}-4(M_{22}M_{23}+M_{21}M_{24}) \cos \omega _{2}\tau _{2}\sqrt{1-\cos ^{2} \tau _{2}\omega _{2}}+2N_{21}(M_{21}+M_{23}) \cos \omega _{2}\tau _{2} \\& \quad {}-2N_{21}(M_{22}+M_{24})\sqrt{1-\cos ^{2}\tau _{2}\omega _{2}}-2N_{22}(M_{21}-M_{23}) \sqrt{1-\cos ^{2}\tau _{2}\omega _{2}} \\& \quad {}+2N_{22}(M_{24}-M_{22})\cos \omega _{2}\tau _{2}=0. \end{aligned}$$

Let $f_{2}(\omega _{2})=\cos \tau _{2}\omega _{2}$, we suppose that $(P_{3})$: $f_{2}(\omega _{2})=\cos \tau _{2}\omega _{2}$ has at least a positive root $\omega _{22}$, which makes Eq. (26) hold. Thus,

21 $$ \tau _{22}^{(i)}=\frac{1}{\omega _{22}} \times \bigl[\arccos \bigl(f_{2}(\omega _{22})\bigr)+2i \pi \bigr],\quad i=0, 1, 2, \ldots . $$

For convenience, we choose

22 $$ \tau _{20}=\min \bigl\{ \tau _{21}^{(i)}, \tau _{22}^{(i)}\bigr\} ,\quad i=0, 1, 2, \ldots , $$

where $\tau _{21}^{(i)}$, $\tau _{21}^{(i)}$ is defined by Eq. (19) and Eq. (21).

Taking the derivative of *λ* with respect to $\tau _{2}$, we obtain

23 $$ \biggl[\frac{d\lambda }{d\tau _{2}} \biggr]^{-1}= \frac{(F_{0}^{\prime }+F_{1}^{\prime })e^{\lambda \tau _{2}}+(F_{2}^{\prime }+F_{3}^{\prime })+(F_{4}^{\prime }+F_{5}^{\prime })e^{-\lambda \tau _{2}}+F_{6}^{\prime }e^{-2\lambda \tau _{2}}}{-\lambda (F_{0}+F_{1})e^{\lambda \tau _{2}}+\lambda (F_{4}+F_{5})e^{-\lambda \tau _{2}}+2\lambda F_{6}e^{-2\lambda \tau _{2}}}- \frac{\tau _{2}}{\lambda }. $$

According to [26], when the hypothesis $(P_{4})$: $\operatorname{Re}[d\lambda /d\tau _{2}]^{-1}_{\tau _{2}=\tau _{20}}\neq 0$ holds, consequently, we have Theorem 2.

### Theorem 2

*For system* (2), *when the hypotheses* $(P_{2})$*–*$(P_{4})$ *hold*, *then* $G^{*}(S^{*}, E^{*}, I^{*}, Q^{*}, R^{*})$ *is locally asymptotically stable when* $\tau _{2}\in [0, \tau _{20})$; *for system* (2) *there appears a Hopf bifurcation at* $G^{*}(S^{*}, E^{*}, I^{*}, Q^{*}, R^{*})$ *when* $\tau _{2}=\tau _{20}$; *system* (2) *becomes unstable when* $\tau _{2}\geqslant \tau _{20}$.

*Case* 4. $\tau _{1}=\tau _{2}=\tau _{*}$. Then Eq. (6) becomes

24 $$ F_{0}(\lambda )+\bigl[F_{1}(\lambda )+F_{2}(\lambda )\bigr]e^{-\lambda \tau _{*}}+\bigl[F_{3}( \lambda )+F_{4}(\lambda )\bigr]e^{-2\lambda \tau _{*}}+ \bigl[F_{5}(\lambda )+F_{6}( \lambda ) \bigr]e^{-3\lambda \tau _{*}}=0. $$

Multiplying $e^{\lambda \tau _{*}}$ on both sides of Eq. (24), then we have

25 $$ F_{0}(\lambda )e^{\lambda \tau _{*}}+ \bigl[F_{1}(\lambda )+F_{2}(\lambda )\bigr]+ \bigl[F_{3}( \lambda )+F_{4}(\lambda ) \bigr]e^{-\lambda \tau _{*}}+\bigl[F_{5}(\lambda )+F_{6}( \lambda )\bigr]e^{-2\lambda \tau _{*}}=0. $$

Substituting $\lambda =i\omega _{3}$ into Eq. (25), we obtain

26 $$ \textstyle\begin{cases} (M_{31}+M_{33})\cos \tau _{*}\omega _{3}+(M_{32}+M_{34})\sin \tau _{*} \omega _{3}+N_{31} \\ \quad =-M_{35}\cos 2\tau _{*}\omega _{3}-M_{36}\sin 2 \tau _{*}\omega _{3}, \\ (M_{34}-M_{32})\cos \tau _{*}\omega _{3}+(M_{31}-M_{33})\sin \tau _{*} \omega _{3}+N_{32} \\ \quad =-M_{36}\cos 2\tau _{*}\omega _{3}+M_{35}\sin 2 \tau _{*}\omega _{3}, \end{cases} $$

with

$$\begin{aligned}& M_{31}=F_{0}^{0}-F_{0}^{2} \omega _{3}^{2}+F_{0}^{4} \omega _{3}^{4}, \\& M_{32}=F_{0}^{3}\omega _{3}^{3}-F_{0}^{1}\omega _{3}-\omega _{3}^{5}, \\& M_{33}=\bigl(F_{3}^{0}+F_{4}^{0} \bigr)-\bigl(F_{3}^{2}+F_{4}^{2} \bigr)\omega _{3}^{2}, \\& M_{34}=\bigl(F_{3}^{1}+F_{4}^{1} \bigr)\omega _{3}-\bigl(F_{3}^{3}+F_{4}^{3} \bigr) \omega _{3}^{3}, \\& M_{35}=\bigl(F_{5}^{0}+F_{6}^{0} \bigr)-\bigl(F_{5}^{2}+F_{6}^{2} \bigr)\omega _{3}^{2}, \\& M_{36}=\bigl(F_{5}^{1}+F_{6}^{1} \bigr)\omega _{3}, \\& N_{31}=\bigl(F_{1}^{0}+F_{2}^{0} \bigr)-\bigl(F_{1}^{2}+F_{2}^{2} \bigr)\omega _{3}^{2}+\bigl(F_{1}^{4}+F_{2}^{4} \bigr) \omega _{3}^{4}, \\& N_{32}=\bigl(F_{1}^{1}+F_{2}^{1} \bigr)\omega _{3}-\bigl(F_{1}^{3}+F_{2}^{3} \bigr) \omega _{3}^{3}. \end{aligned}$$

Squaring both sides of the two equations in Eq. (26), and summing them, we have

27 $$\begin{aligned}& (M_{31}+M_{33})^{2}\cos ^{2}\tau _{*}\omega _{3}+(M_{32}+M_{34})^{2} \sin ^{2}\tau _{*}\omega _{3} \\& \quad {}+2(M_{31}+M_{33}) (M_{32}+M_{34}) \cos \tau _{*}\omega _{3}\sin \tau _{*}\omega _{3} \\& \quad {}+2N_{31}(M_{31}+M_{33})\cos \tau _{*}\omega _{3}+2N_{31}(M_{32}+M_{34}) \sin \tau _{*}\omega _{3} \\& \quad {}+(M_{34}-M_{32})^{2}\cos ^{2}\tau _{*} \omega _{3}+N_{31}^{2}+N_{32}^{2} \\& \quad {}+(M_{31}-M_{33})^{2}\sin ^{2}\tau _{*}\omega _{3}+2N_{32}(M_{34}-M_{32}) \cos \tau _{*}\omega _{3} \\& \quad {}+2N_{32}(M_{31}-M_{33})\sin \tau _{*}\omega _{3}-M_{35}^{2}-M_{36}^{2} \\& \quad {}+2(M_{34}-M_{32}) (M_{31}-M_{33}) \cos \tau _{*}\omega _{3}\sin \tau _{*} \omega _{3}=0. \end{aligned}$$

We have $\sin \tau _{*}\omega _{3}=\pm \sqrt{1-\cos ^{2}\tau _{*}\omega _{3}}$.

1. If $\sin \tau _{*}\omega _{3}=\sqrt{1-\cos ^{2}\tau _{*}\omega _{3}}$, from Eq. (27), we can get
  28 $$\begin{aligned}& (M_{31}+M_{33})^{2}\cos ^{2}\tau _{*}\omega _{3}+(M_{32}+M_{34})^{2} \bigl(1- \cos ^{2}\tau _{*}\omega _{3}\bigr) \\& \quad {}+N_{31}^{2}+N_{32}^{2}-M_{35}^{2}-M_{36}^{2} \\& \quad {}+2(M_{31}+M_{33}) (M_{32}+M_{34}) \cos \tau _{*}\omega _{3}\sqrt{1- \cos ^{2} \tau _{*}\omega _{3}} \\& \quad {}+2N_{31}(M_{31}+M_{33})\cos \tau _{*} \omega _{3} \\& \quad {}+(M_{34}-M_{32})^{2}\cos ^{2}\tau _{*}\omega _{3}+(M_{31}-M_{33})^{2} \bigl(1- \cos ^{2}\tau _{*}\omega _{3}\bigr) \\& \quad {}+2N_{32}(M_{34}-M_{32})\cos \tau _{*} \omega _{3} \\& \quad {}+2N_{32}(M_{31}-M_{33})\sqrt{1-\cos ^{2}\tau _{*}\omega _{3}} \\& \quad {}+2(M_{34}-M_{32}) (M_{31}-M_{33}) \cos \tau _{*}\omega _{3}\sqrt{1-\cos ^{2} \tau _{*}\omega _{3}} \\& \quad {}+2N_{31}(M_{32}+M_{34})\sqrt{1-\cos ^{2}\tau _{*}\omega _{3}}=0. \end{aligned}$$

Let $f_{3}(\omega _{3})=\cos \tau _{*}\omega _{3}$, and we suppose that $(P_{5})$: $f_{3}(\omega _{3})=\cos \tau _{*}\omega _{3}$ has at least a positive root $\omega _{31}$, which makes Eq. (28) hold. Thus,

29 $$ \tau _{*1}^{(i)}=\frac{1}{\omega _{31}} \times \bigl[\arccos \bigl(f_{3}(\omega _{31})\bigr)+2i \pi \bigr],\quad i=0, 1, 2, \ldots . $$

(2) If $\sin \tau _{*}\omega _{3}=-\sqrt{1-\cos ^{2}\tau _{*}\omega _{3}}$, after calculation, we have
  30 $$\begin{aligned}& (M_{31}+M_{33})^{2}\cos ^{2}\tau _{*}\omega _{3}+(M_{32}+M_{34})^{2} \bigl(1- \cos ^{2}\tau _{*}\omega _{3} \bigr)+N_{31}^{2}+N_{32}^{2}-M_{35}^{2}-M_{36}^{2} \\& \quad {}-2(M_{31}+M_{33}) (M_{32}+M_{34}) \cos \tau _{*}\omega _{3}\sqrt{1- \cos ^{2} \tau _{*}\omega _{3}} \\& \quad {}+2N_{31}(M_{31}+M_{33})\cos \tau _{*} \omega _{3} \\& \quad {}+(M_{34}-M_{32})^{2}\cos ^{2}\tau _{*}\omega _{3}+(M_{31}-M_{33})^{2} \bigl(1- \cos ^{2}\tau _{*}\omega _{3}\bigr) \\& \quad {}+2N_{32}(M_{34}-M_{32})\cos \tau _{*} \omega _{3} \\& \quad {}-2N_{32}(M_{31}-M_{33})\sqrt{1-\cos ^{2}\tau _{*}\omega _{3}} \\& \quad {}-2(M_{34}-M_{32}) (M_{31}-M_{33}) \cos \tau _{*}\omega _{3}\sqrt{1-\cos ^{2} \tau _{*}\omega _{3}} \\& \quad {}-2N_{31}(M_{32}+M_{34})\sqrt{1-\cos ^{2}\tau _{*}\omega _{3}}=0. \end{aligned}$$

Let $f_{4}(\omega _{3})=\cos \tau _{*}\omega _{3}$, and we suppose that $(P_{6})$: $f_{4}(\omega _{3})=\cos \tau _{*}\omega _{3}$ has at least a positive root $\omega _{32}$, which makes Eq. (30) hold. Thus,

31 $$ \tau _{*2}^{(i)}=\frac{1}{\omega _{32}} \times \bigl[\arccos \bigl(f_{4}(\omega _{32})\bigr)+2i \pi \bigr],\quad i=0, 1, 2, \ldots . $$

Define

32 $$ \tau _{*0}=\min \bigl\{ \tau _{*1}^{(i)}, \tau _{*2}^{(i)}\bigr\} ,\quad i=0, 1, 2, \ldots , $$

where $\tau _{*1}^{(i)}$ and $\tau _{*2}^{(i)}$ are defined by Eq. (29) and Eq. (31), respectively.

Then after taking the derivative to $\tau _{*}$ of *λ*, we can get

33 $$\begin{aligned} \biggl[\frac{d\lambda }{d\tau _{*}} \biggr]^{-1} =& \frac{F_{0}^{\prime }(\lambda )e^{\lambda \tau _{*}}+[F_{1}^{\prime }(\lambda ) +F_{2}^{\prime }(\lambda )]+[F_{3}^{\prime }(\lambda )+F_{4}^{\prime }(\lambda )] e^{-\lambda \tau _{*}}+(F_{5}^{\prime }+F_{6}^{\prime })(\lambda )e^{-2\lambda \tau _{*}}}{-\lambda F_{0}(\lambda )e^{\lambda \tau _{*}}+\lambda [F_{3}(\lambda )+F_{4}(\lambda )]e^{-\lambda \tau _{*}}+2\lambda (F_{5}(\lambda )+ F_{6}(\lambda ))e^{-2\lambda \tau _{*}}} \\ &{}- \frac{\tau _{*}}{\lambda }. \end{aligned}$$

According to [26], when the hypothesis $(P_{7})$: $\operatorname{Re}[d\lambda /d\tau _{*}]^{-1}_{\tau _{*}=\tau _{*0}}\neq 0$ holds, the virus-existence equilibrium $G^{*}(S^{*}, E^{*}, I^{*}, Q^{*}, R^{*})$ is locally asymptotically stable. Therefore, Theorem 3 can be obtained.

### Theorem 3

*For system* (2), *when the hypotheses* $(P_{5})$*–*$(P_{7})$ *hold*, *then* $G^{*}(S^{*}, E^{*}, I^{*}, Q^{*}, R^{*})$ *is locally asymptotically stable when* $\tau _{*}\in [0, \tau _{*0})$; *system* (2) *undergoes a Hopf bifurcation at* $G^{*}(S^{*}, E^{*}, I^{*}, Q^{*}, R^{*})$ *when* $\tau _{*}=\tau _{*0}$; *system* (2) *becomes unstable when* $\tau _{*}\geqslant \tau _{*0}$.

*Case* 5. $\tau _{1}>\tau _{2}$, $\tau _{2}\in (0, \tau _{20})$. Then Eq. (6) becomes

34 $$ \bigl[F_{0}(\lambda )+F_{2}(\lambda )+F_{3}(\lambda )+F_{4}(\lambda )+F_{5}( \lambda )+F_{6}(\lambda )\bigr]+\bigl[F_{1}(\lambda )+F_{3}(\lambda )+F_{5}( \lambda )\bigr]e^{-\lambda \tau _{1}}=0. $$

Let $\lambda =i\omega _{4}$. Separating the real and imaginary parts, we obtain

35 $$ \textstyle\begin{cases} M_{41}\cos \tau _{1}\omega _{4}+M_{42}\sin \tau _{1}\omega _{4}=-N_{41}, \\ M_{42}\cos \tau _{1}\omega _{4}-M_{41}\sin \tau _{1}\omega _{4}=-N_{42}, \end{cases} $$

with

$$\begin{aligned}& M_{41}=\bigl(F_{1}^{0}+F_{3}^{0}+F_{5}^{0} \bigr)-\bigl(F_{1}^{2}+F_{3}^{2}+F_{5}^{2} \bigr) \omega _{1}^{2}+F_{1}^{4} \omega _{1}^{4}, \\& M_{42}=\bigl(F_{1}^{1}+F_{3}^{1}+F_{5}^{1} \bigr)\omega _{1}-\bigl(F_{1}^{3}+F_{3}^{3} \bigr) \omega _{1}^{3}, \\& N_{41}=\bigl(F_{0}^{0}+F_{2}^{0}+F_{3}^{0}+F_{4}^{0}+F_{5}^{0}+F_{6}^{0} \bigr)-\bigl(F_{0}^{2}+F_{2}^{2}+F_{3}^{2}+F_{4}^{2}+F_{5}^{2}+F_{6}^{2} \bigr) \omega _{1}+\bigl(F_{0}^{4}+F_{2}^{4} \bigr)\omega _{1}, \\& N_{42}=\bigl(F_{0}^{0}+F_{2}^{0}+F_{3}^{0}+F_{4}^{0}+F_{5}^{0}+F_{6}^{0} \bigr) \omega _{1}-\bigl(F_{0}^{3}+F_{2}^{3}+F_{3}^{3}+F_{4}^{3} \bigr)\omega _{1}^{3}. \end{aligned}$$

Squaring both sides of two equations in Eq. (35), and summing them, Eq. (36) can be obtained:

36 $$\begin{aligned} M_{41}^{2}+M_{42}^{2}=N_{41}^{2}+N_{42}^{2}. \end{aligned}$$

We suppose that $(P_{8})$: Eq. (36) has at least one positive real root $\omega _{40}$. Solving Eq. (36), we obtain

37 $$ \tau _{1}^{(k)}=\frac{1}{\omega _{40}} \times \biggl[\arccos \frac{M_{41}N_{41}+M_{42}N_{42}}{M_{41}^{2}+M_{42}^{2}}+2k\pi \biggr],\quad k=0, 1, 2, \ldots . $$

For convenience, we define

38 $$ \tau _{10}=\min \bigl\{ \tau _{1}^{(k)}, k=0, 1, 2, \ldots \bigr\} , $$

where $\tau _{1}^{(k)}$ is defined by Eq. (37).

Taking the derivative to $\tau _{1}$ of *λ*, we can get

39 $$\begin{aligned}& \biggl[\frac{d\lambda }{d\tau _{1}} \biggr]^{-1} \\& \quad = \frac{[F_{0}^{\prime }(\lambda )+F_{2}^{\prime }(\lambda )+F_{3}^{\prime }(\lambda )+F_{4}^{\prime }(\lambda )+F_{5}^{\prime }(\lambda )+F_{6}^{\prime }(\lambda )]+[F_{1}^{\prime }(\lambda )+F_{3}^{\prime }(\lambda )+F_{5}^{\prime }(\lambda )]e^{-\lambda \tau _{1}}}{\lambda [F_{1}(\lambda )+F_{3}(\lambda )+F_{5}^{\prime }(\lambda )]e^{-\lambda \tau _{1}}} \\& \qquad {}- \frac{\tau _{1}}{\lambda }. \end{aligned}$$

According to [26], when the hypothesis $(P_{9})$: $\operatorname{Re}[d\lambda /d\tau _{1}]^{-1}_{\tau _{1}=\tau _{10}}\neq 0$ holds, the virus-existence equilibrium $G^{*}(S^{*}, E^{*}, I^{*}, Q^{*}, R^{*})$ is locally asymptotically stable. So, we have Theorem 4.

### Theorem 4

*For system* (2), *when the hypotheses* $(P_{8})$*–*$(P_{9})$ *is true*, *then* $G^{*}(S^{*}, E^{*}, I^{*}, Q^{*}, R^{*})$ *is locally asymptotically stable when* $\tau _{1}\in (0, \tau _{10})$; *system* (2) *undergoes a Hopf bifurcation at* $G^{*}(S^{*}, E^{*}, I^{*}, Q^{*}, R^{*})$ *when* $\tau _{1}=\tau _{10}$; *once* $\tau _{1}$ *exceeds* $\tau _{10}$, *system* (2) *becomes unstable*.

## Direction and stability of Hopf bifurcation

It is important for controlling chaos to research direction and stability of the Hopf bifurcation. In this section, we use manifold theory in [27] to discuss direction and stability of the Hopf bifurcation of system (2). We assume that $\tau _{2}^{*}<\tau _{1}^{*}$, where $\tau _{2}^{*}\in (0, \tau _{20})$. Let $\tau _{1}=\tau _{1}^{*}+\omega (\omega \in \text{R})$, $\rho _{1}=S(\tau _{1} t)$, $\rho _{2}=E(\tau _{1} t)$, $\rho _{3}=I(\tau _{1} t)$, $\rho _{4}=Q(\tau _{1} t)$, $\rho _{5}=R(\tau _{1} t)$. System (2) becomes

40 $$ \dot{\rho }(t)=L_{\omega }(\rho _{t})+F( \omega , \rho _{t}), $$

where $\rho (t)=(\rho _{1}, \rho _{2}, \rho _{3}, \rho _{4}, \rho _{5})^{T} \in C=C([-1, 0], \text{R}^{5})$ and $L_{\omega }$: $C\rightarrow \text{R}^{5}$ and *F*: $\text{R}\times C\rightarrow \text{R}^{5}$ are defined as

41 $$ L_{\omega }\phi =\bigl(\tau _{1}^{*}+ \omega \bigr) \biggl(A^{\prime }\phi (0)+B^{ \prime }\phi \biggl(- \frac{\tau _{2}^{*}}{\tau _{1}^{*}} \biggr)+C^{ \prime }\phi (-1) \biggr) $$

and

42 $$ F(\omega , \phi )=\bigl(\tau _{1}^{*}+ \omega \bigr)[F_{1}, F_{2}, 0, 0, 0]^{T}, $$

with

$$\begin{array}{c} A^{\prime }=\left(\begin{array}{ccccc} a_{11} & a_{12} & a_{13} & 0 & 0\\ a_{21} & a_{22} & a_{23} & 0 & 0\\ 0 & 0 & a_{33} & 0 & 0\\ 0 & a_{42} & a_{43} & a_{44} & 0\\ 0 & 0 & 0 & 0 & a_{55} \end{array}\right),\;B^{\prime }=\left(\begin{array}{ccccc} 0 & 0 & 0 & 0 & 0\\ 0 & b_{22} & 0 & 0 & 0\\ 0 & b_{32} & 0 & 0 & 0\\ 0 & 0 & 0 & 0 & 0\\ 0 & 0 & 0 & 0 & 0 \end{array}\right),\\ C^{\prime }=\left(\begin{array}{ccccc} 0 & 0 & 0 & 0 & 0\\ 0 & c_{22} & 0 & 0 & 0\\ 0 & 0 & c_{33} & 0 & 0\\ 0 & 0 & 0 & c_{44} & 0\\ 0 & c_{52} & c_{53} & c_{54} & 0 \end{array}\right), \end{array}$$

and

$$\begin{aligned}& F_{1}=-\beta _{2}\phi _{1}(0)\phi _{2}(0)-\beta _{1}\phi _{1}(0) \phi _{3}(0)+\cdots , \\& F_{2}=\beta _{2}\phi _{1}(0)\phi _{2}(0)+\beta _{1}\phi _{1}(0) \phi _{3}(0)+\cdots . \end{aligned}$$

By the Riesz representation theorem, $\eta (\vartheta , \omega )$ can be defined, and $\vartheta \in [-1, 0)$. Thus,

43 $$ L_{\omega }\phi = \int _{-1}^{0}d\eta (\vartheta , \omega )\phi ( \vartheta ). $$

For convenience, we choose

44 $$ \eta (\vartheta ,\omega )= \textstyle\begin{cases} (\tau _{1}^{*}+\omega )(A^{\prime }+B^{\prime }+C^{\prime }), \qquad \vartheta =0, \\ (\tau _{1}^{*}+\omega )(B^{\prime }+C^{\prime }), \qquad \vartheta \in [-\frac{\tau _{2}^{*}}{\tau _{1}^{*}}, 0), \\ (\tau _{1}^{*}+\omega )(C^{\prime }), \qquad \vartheta \in (-1, -\frac{\tau _{2}^{*}}{\tau _{1}^{*}}), \\ 0, \qquad \vartheta =-1, \end{cases} $$

with $\varrho (\vartheta )$ the Dirac delta function.

For $\phi \in C([-1,0], R^{5})$, define

$$ A(\omega )\phi =\textstyle\begin{cases} \frac{d\phi (\vartheta )}{d\vartheta }, \quad -1\leq \vartheta < 0, \\ \int _{-1}^{0}d\eta (\vartheta ,\omega )\phi (\vartheta ), \quad \vartheta =0, \end{cases} $$

and

$$ R(\omega )\phi =\textstyle\begin{cases} 0, \quad -1\leq \vartheta < 0, \\ F(\omega ,\phi ), \quad \vartheta =0. \end{cases} $$

Then system (2) is equivalent to

45 $$ \dot{\rho }(t)=A(\omega )\rho _{t}+R(\omega )\rho _{t}. $$

For $\psi \in C^{1}([0, 1], (R^{5})^{*})$, define

$$ A^{*}(\psi )=\textstyle\begin{cases} -\frac{d\psi (s)}{ds}, \quad 0< s\leq 1, \\ \int _{-1}^{0}d{\eta }^{T}(s,0)\psi (-s), \quad s=0, \end{cases} $$

and the bilinear inner form for $A(0)$ and $A^{*}$

46 $$ \bigl\langle \psi (s),\phi (\vartheta )\bigr\rangle =\bar{\psi }(0)\phi (0)- \int _{\vartheta =-1}^{0} \int _{\varsigma =0}^{\vartheta }\bar{\psi }( \varsigma -\vartheta )\,d\eta (\vartheta )\phi (\varsigma )\,d\varsigma , $$

where $\eta (\vartheta )=\eta (\vartheta , 0)$.

Let $n(\vartheta )=(1, n_{2}, n_{3}, n_{4}, n_{5})^{T}e^{i\tau _{1}^{*} \omega _{1}^{*}\vartheta }$ and $n^{*}(s)=D(1, n_{2}^{*}, n_{3}^{*}, n_{4}^{*}, n_{5}^{*})^{T}e^{i \tau _{1}^{*}\omega _{1}^{*}s}$. Based on the definitions of $A(0)$ and $A^{*}(0)$, we can obtain

$$\begin{aligned}& n_{2}=\frac{a_{21}+n_{3}a_{23}}{i\omega _{1}^{*}-a_{22}-b_{22}e^{-i\tau _{1}^{*}\omega _{1}^{*}}-c_{22}e^{-i\tau _{2}^{*}\omega _{1}^{*}}}, \\& n_{3}=\frac{a_{21}b_{32}e^{-i\tau _{1}^{*}\omega _{1}^{*}}}{\chi -a_{23}b_{32}e^{-i\tau _{1}^{*}\omega _{1}^{*}}}, \\& n_{4}=\frac{a_{42}n_{2}+a_{43}n_{3}}{i\omega _{1}^{*}-a_{44}-c_{44}e^{-i\tau _{2}^{*}\omega _{1}^{*}}}, \\& n_{5}=\frac{\chi _{2}}{i\omega _{1}^{*}-a_{55}}, \\& n_{2}^{*}=-\frac{i\omega _{1}^{*}+a_{11}}{a_{21}}, \\& n_{3}^{*}=- \frac{a_{23}n_{2}^{*}}{i\tau _{1}^{*}\omega _{1}^{*}+a_{33}+c_{33}e^{-i\tau _{2}^{*}\omega _{1}^{*}}}, \\& n_{4}^{*}=0, \\& n_{5}^{*}=0, \end{aligned}$$

where

$$\begin{aligned}& \chi _{1}=\bigl(i\omega _{1}^{*}-a_{22}-b_{22}e^{-i\tau _{1}^{*}\omega _{1}^{*}}-c_{22}e^{-i \tau _{2}^{*}\omega _{1}^{*}} \bigr) \bigl(i\omega _{1}^{*}-a_{33}-b_{32}e^{-i \tau _{1}^{*}\omega _{1}^{*}}-c_{33}e^{-i\tau _{2}^{*}\omega _{1}^{*}} \bigr), \\& \chi _{2}=c_{52}n_{2}e^{-i\tau _{2}^{*}\omega _{1}^{*}}+c_{53}n_{3}e^{-i \tau _{2}^{*}\omega _{1}^{*}}+c_{54}n_{4}e^{-i\tau _{2}^{*}\omega _{1}^{*}}. \end{aligned}$$

Then we have

$$\begin{aligned} \bar{D} =&\bigl[1+n_{2}\bar{n}_{2}^{*}+n_{3} \bar{n}_{3}^{*}+n_{4}\bar{n}_{4}^{*}+n_{5} \bar{n}_{5}^{*}+\tau _{1}^{*}e^{-i\tau _{1}^{*}\omega _{1}^{*}}n_{2} \bigl(b_{22} \bar{n}_{2}^{*}+b_{32} \bar{n}_{3}^{*}\bigr) \\ &{}+\tau _{2}^{*}e^{-i\tau _{2}^{*}\omega _{1}^{*}}n_{2} \bigl(c_{22}\bar{n}_{2}^{*}+c_{52} \bar{n}_{5}^{*}\bigr)+\tau _{2}^{*}e^{-i\tau _{2}^{*}\omega _{1}^{*}}n_{4} \bigl(c_{33} \bar{n}_{3}^{*}+c_{53} \bar{n}_{5}^{*}\bigr) \\ &{}+\tau _{2}^{*}e^{-i\tau _{2}^{*}\omega _{1}^{*}}n_{4} \bigl(c_{44}\bar{n}_{4}^{*}+c_{54} \bar{n}_{5}^{*}\bigr)\bigr]^{-1}. \end{aligned}$$

Next, we can obtain $g_{20}$, $g_{11}$, $g_{02}$ and $g_{21}$ by means of the method in [13]:

$$\begin{aligned}& g_{20}=2\tau _{1}^{*}\bar{D}\bigl( \bar{n}_{2}^{*}-1\bigr)\bigl[\beta _{2}(n_{2}+ \bar{n}_{2})+\beta _{1}(n_{3}+ \bar{n}_{3}))\bigr], \\& g_{11}=2\tau _{1}^{*}\bar{D}\bigl( \bar{n}_{2}^{*}-1\bigr)\bigl[\beta _{2}(n_{2}+ \bar{n}_{2})+\beta _{1}(n_{3}+ \bar{n}_{3}))\bigr], \\& g_{02}=2\tau _{1}^{*}\bar{D}\bigl( \bar{n}_{2}^{*}-1\bigr)[\beta _{2} \bar{n}_{2}+ \beta _{1}\bar{n}_{3}], \\& g_{21}=2\tau _{1}^{*}\bar{D}\bigl( \bar{n}_{2}^{*}-1\bigr) \biggl[\beta _{2} \biggl(W_{11}^{(2)}(0)+\frac{1}{2}W_{20}^{(2)}(0)+n_{2}W_{11}^{(1)}(0)+ \frac{\bar{n}_{2}}{2}W_{20}^{(1)}(0) \biggr) \\& \hphantom{g_{21}=}{}+\beta _{1} \biggl(W_{11}^{(3)}(0)+ \frac{1}{2}W_{20}^{(3)}(0)+n_{2}W_{11}^{(1)}(0)+ \frac{\bar{n}_{3}}{2}W_{20}^{(1)}(0) \biggr) \biggr], \end{aligned}$$

with

$$\begin{aligned}& W_{20}(\vartheta )=\frac{ig_{20}n(0)}{\tau _{1}^{*}\omega _{1}^{*}}e^{i \tau _{1}^{*}\omega _{1}^{*}\vartheta }+ \frac{i\bar{g}_{02}\bar{n}(0)}{3\tau _{1}^{*}\omega _{1}^{*}}e^{-i \tau _{1}^{*}\omega _{1}^{*}\vartheta }+U_{1}e^{2i\tau _{1}^{*}\omega _{1}^{*} \vartheta }, \\& W_{11}(\vartheta )=- \frac{ig_{11}n(0)}{\tau _{1}^{*}\omega _{1}^{*}}e^{i\tau _{1}^{*} \omega _{1}^{*}\vartheta }+ \frac{i\bar{g}_{11}\bar{n}(0)}{\tau _{1}^{*}\omega _{1}^{*}}e^{-i \tau _{1}^{*}\omega _{1}^{*}\vartheta }+U_{2}. \end{aligned}$$

$U_{1}$ and $U_{2}$ can be computed by

$$\begin{array}{c} U_{1}=2{\left(\begin{array}{ccccc} a_{11}^{*} & -a_{12} & -a_{13} & 0 & 0\\ -a_{21} & a_{22}^{*} & 0 & 0 & 0\\ 0 & -b_{32}e^{-i\tau_{1}^{*}\omega_{1}^{*}} & a_{33}^{*} & 0 & 0\\ 0 & -a_{42} & -a_{43} & a_{44}^{*} & 0\\ 0 & -c_{52}e^{-i\tau_{2}^{*}\omega_{1}^{*}} & -c_{53}e^{-i\tau_{2}^{*}\omega_{1}^{*}} & -c_{54}e^{-i\tau_{2}^{*}\omega_{1}^{*}} & a_{55}^{*} \end{array}\right)}^{-1}\times \left(\begin{array}{c} U_{1}^{\left(1\right)}\\ U_{1}^{\left(2\right)}\\ 0\\ 0\\ 0 \end{array}\right),\\ U_{2}=-{\left(\begin{array}{ccccc} a_{11} & a_{12} & a_{13} & 0 & 0\\ a_{21} & a_{22}+b_{22}+c_{22} & a_{23} & 0 & 0\\ 0 & b_{32} & a_{33}+c_{33} & 0 & 0\\ 0 & a_{42} & a_{43} & a_{44}+c_{44} & 0\\ 0 & c_{52} & c_{53} & c_{54} & a_{55} \end{array}\right)}^{-1}\times \left(\begin{array}{c} U_{2}^{\left(1\right)}\\ U_{2}^{\left(2\right)}\\ 0\\ 0\\ 0 \end{array}\right), \end{array}$$

where

$$\begin{aligned}& a_{11}^{*}=2i\omega _{1}^{*}-a_{11}, \\& a_{22}^{*}=2i\omega _{1}^{*}-a_{22}-b_{22}e^{-i\tau _{1}^{*}\omega _{1}^{*}}-c_{22}e^{-i \tau _{2}^{*}\omega _{1}^{*}}, \\& a_{33}^{*}=2i\omega _{1}^{*}-a_{33}-c_{33}e^{-i\tau _{2}^{*}\omega _{1}^{*}}, \\& a_{44}^{*}=2i\omega _{1}^{*}-a_{44}-c_{44}e^{-i\tau _{2}^{*}\omega _{1}^{*}}, \\& a_{55}^{*}=2i\omega _{1}^{*}-a_{55}, \end{aligned}$$

and

$$\begin{aligned}& U_{1}^{(1)}=-\beta _{2}n_{2}- \beta _{1}n_{3}, \\& U_{1}^{(2)}=\beta _{2}n_{2}+ \beta _{1}n_{3}, \\& U_{2}^{(1)}=-\beta _{2}(n_{2}+ \bar{n}_{2})-\beta _{1}(n_{3}+ \bar{n}_{3}), \\& U_{2}^{(2)}=\beta _{2}(n_{2}+ \bar{n}_{2})+\beta _{1}(n_{3}+ \bar{n}_{3}). \end{aligned}$$

Then we can obtain

47 $$ \begin{aligned} &C_{1}(0)=\frac{i}{2\tau _{1}^{*}\omega _{1}^{*}} \biggl(g_{11}g_{20}-2 \vert g_{11} \vert ^{2}- \frac{ \vert g_{02} \vert ^{2}}{3} \biggr)+\frac{g_{21}}{2}, \\ &\mu _{2} =- \frac{\operatorname{Re}\{C_{1}(0)\}}{\operatorname{Re}\{\lambda ^{\prime }(\tau _{1}^{*})\}}, \\ &\beta _{2}=2{\operatorname{Re}\bigl\{ C_{1}(0)\bigr\} }, \\ &T_{2}=- \frac{\text{Im}\{C_{1}(0)\}+\mu _{2}\text{Im}\{\lambda ^{\prime }(\tau _{1}^{*})\}}{\tau _{1}^{*}\omega _{1}^{*}}. \end{aligned} $$

Thus, we have Theorem 4 about the Hopf bifurcation at $\tau _{1}^{*}$.

### Theorem 5

*For system* (2), *the following results hold*. *If* $\mu _{2}>0$ ($\mu _{2}<0$), *then the Hopf bifurcation is supercritical* (*subcritical*); *if* $\beta _{2}<0$ ($\beta _{2}>0$), *then the bifurcating periodic solutions are stable* (*unstable*); *if* $T_{2}>0$ ($T_{2}<0$), *then the period of the bifurcating periodic solutions increase* (*decrease*).

## Numerical simulations

Choosing $\lambda =0.5$, $\beta _{1}=0.64$, $\beta _{2}=0.185$, $\beta _{3}=0.05$, $\mu =0.05$, $q_{1}=0.12$, $\alpha =0.28$, $\kappa =0.15$, $d_{1}=0.3$, $q_{2}=0.99$, $r=0.11$, $d_{2}=0.25$, $q=0.14$, we use Matlab to verify the correctness of above theorems. Then system (2) takes the form

48 $$ \textstyle\begin{cases} \frac{dS(t)}{dt}=0.5-0.05S(t)-(0.185E(t)+0.64I(t)+0.05)S(t), \\ \frac{dE(t)}{dt}=(0.185E(t)+0.64I(t)+0.05)S(t)-0.17E(t) \\ \hphantom{\frac{dE(t)}{dt}=}{}-0.28E(t- \tau _{1})-0.15E(t-\tau _{2}), \\ \frac{dI(t)}{dt}=0.28E(t-\tau _{1})-1.34I(t)-0.11I(t-\tau _{2}), \\ \frac{dQ(t)}{dt}=0.12E(t)+0.99I(t)-0.3Q(t)-0.14Q(t-\tau _{2}), \\ \frac{dR(t)}{dt}=0.15E(t-\tau _{2})+0.11I(t-\tau _{2})+0.14Q(t- \tau _{2})-0.05R(t), \end{cases} $$

from which we can obtain $G^{*}(1.58, 0.7016, 0.1353, 0.4959, 3.793)$.

**Figure 1:**
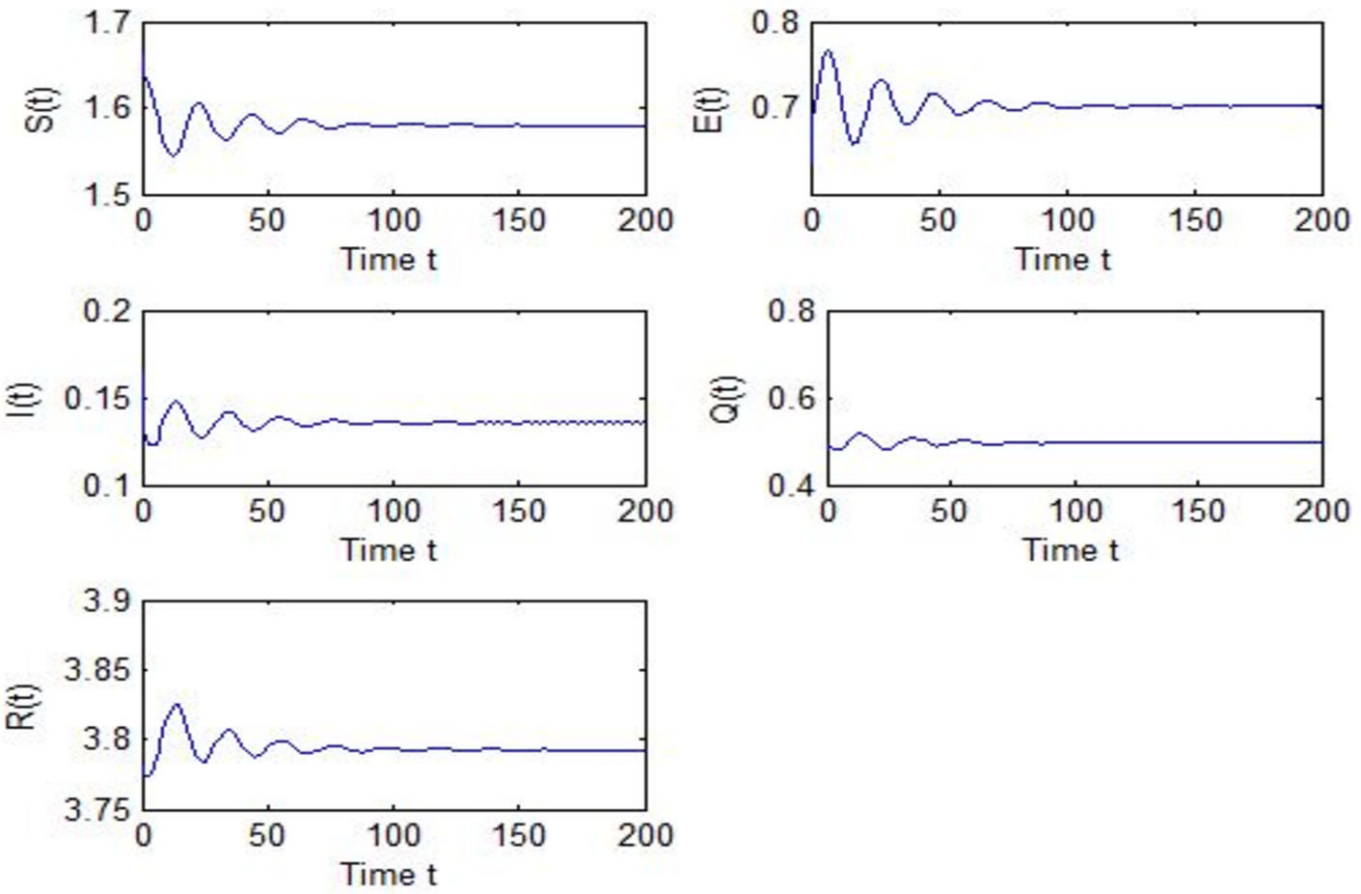
Evolutions of *S*, *E*, *I*, *Q*, *R* for $\tau _{1}=6.037<\tau _{10}$ along with time *t*

**Figure 2:**
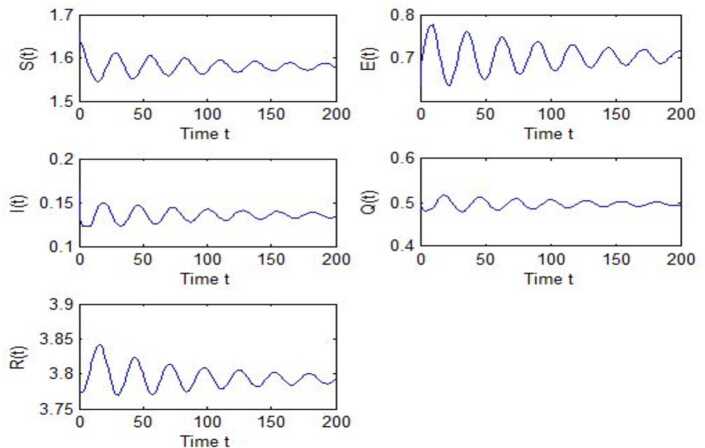
Evolutions of *S*, *E*, *I*, *Q*, *R* for $\tau _{1}=9.7291>\tau _{10}$ along with time *t*

**Figure 3:**
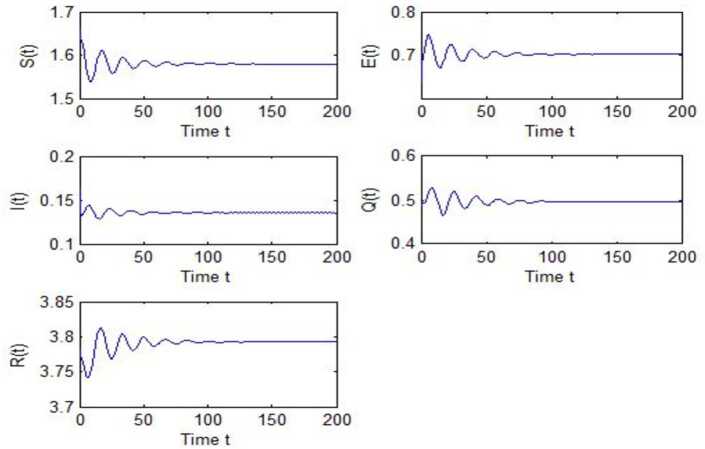
Evolutions of *S*, *E*, *I*, *Q*, *R* for $\tau _{2}=5.1264<\tau _{20}$ along with time *t*

**Figure 4:**
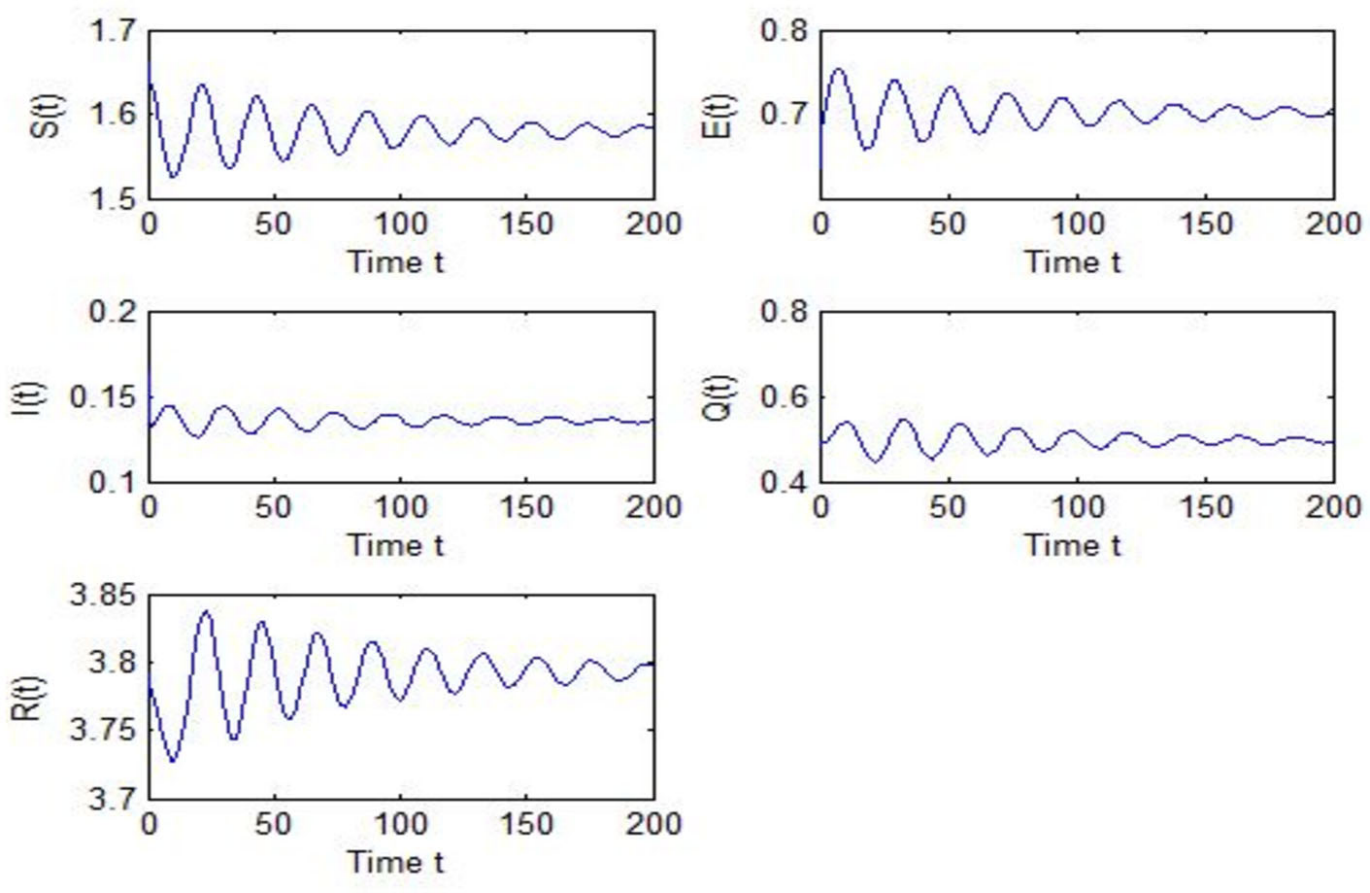
Evolutions of *S*, *E*, *I*, *Q*, *R* for $\tau _{2}=8.9951>\tau _{20}$ along with time *t*

**Figure 5:**
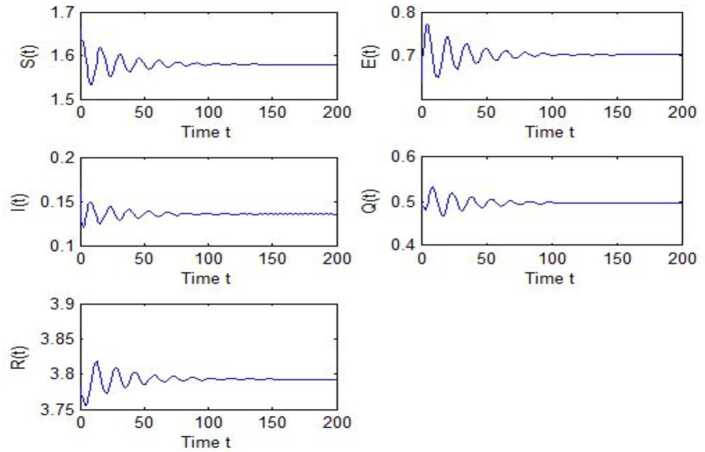
Evolutions of *S*, *E*, *I*, *Q*, *R* for $\tau _{*}=2.7471<\tau _{*0}$ along with time *t*

**Figure 6:**
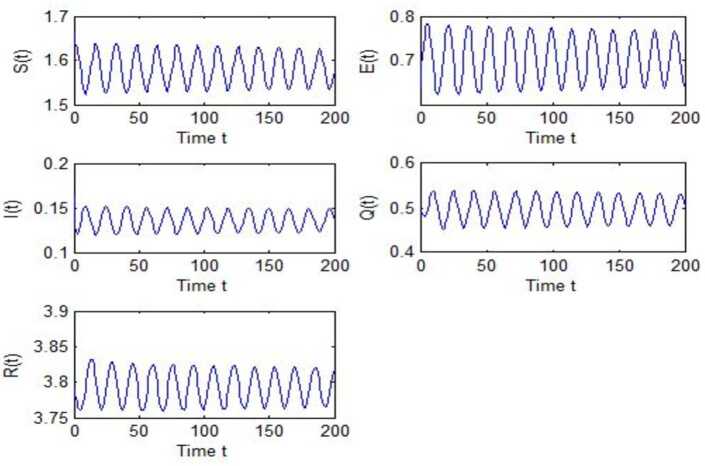
Evolutions of *S*, *E*, *I*, *Q*, *R* for $\tau _{*}=3.1951>\tau _{*0}$ along with time *t*

**Figure 7:**
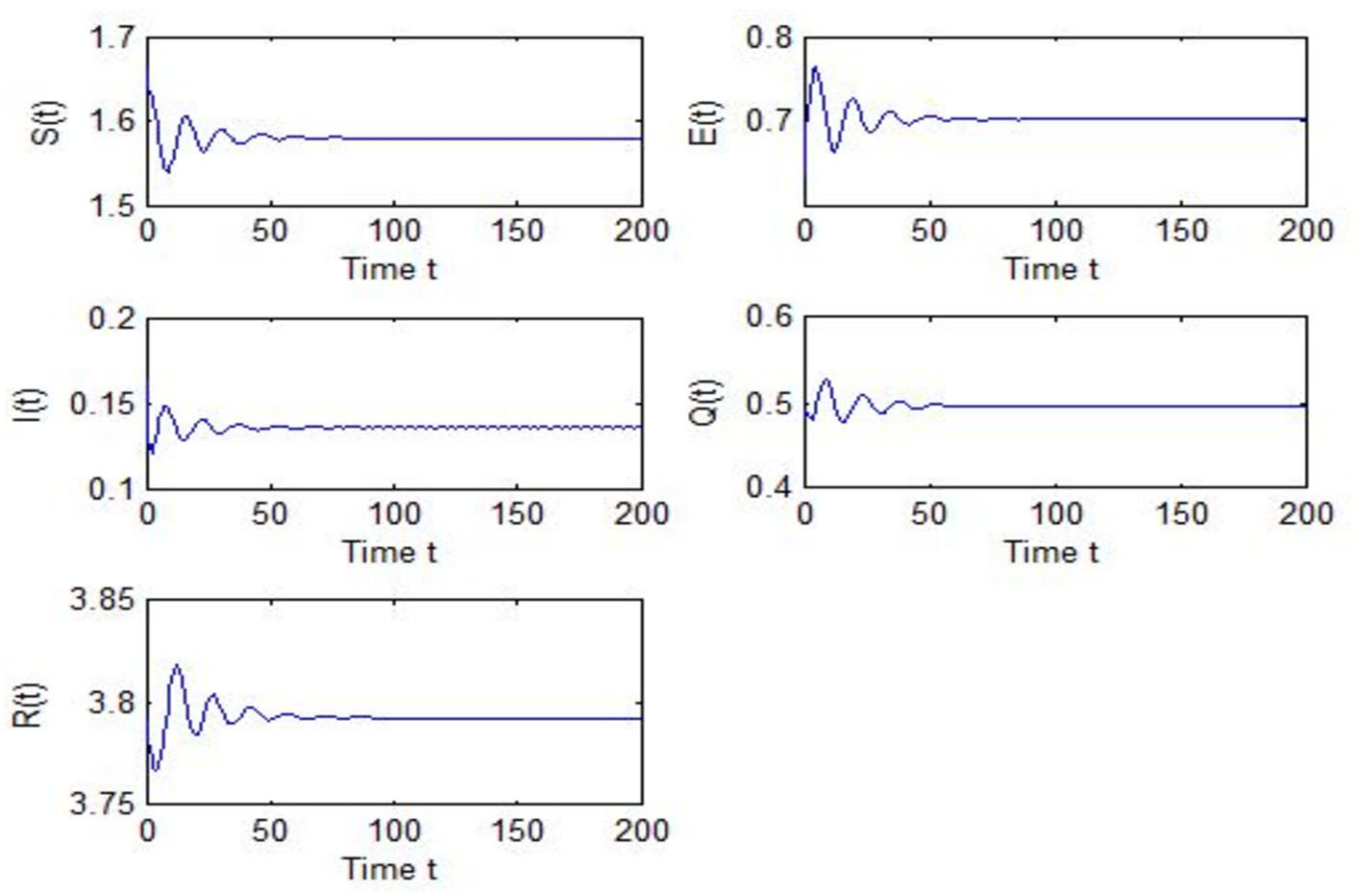
Evolutions of *S*, *E*, *I*, *Q*, *R* for $\tau _{1}=2.5966<\tau _{10}$, $\tau _{2}=2.299$ along with time *t*

**Figure 8:**
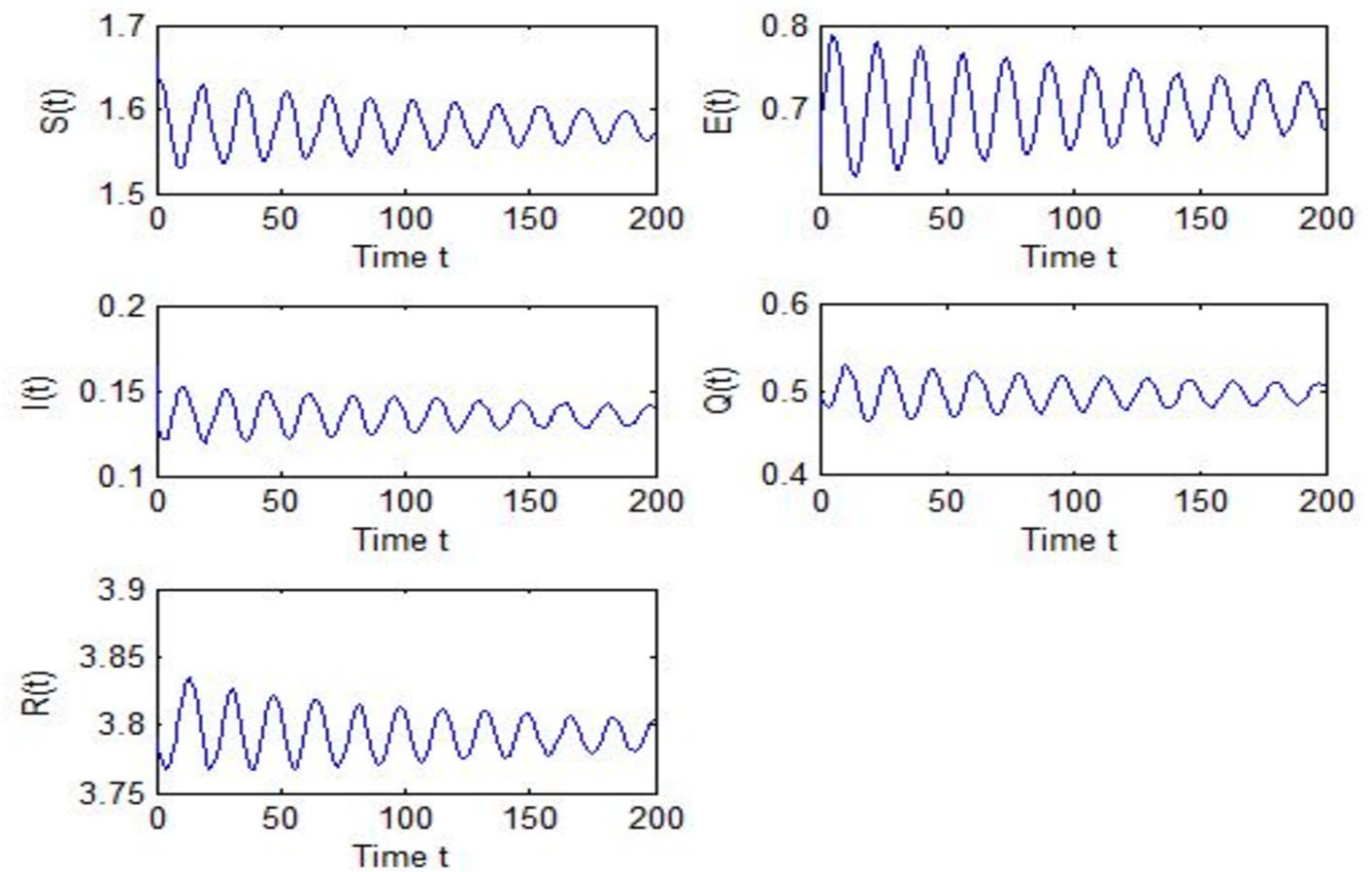
Evolutions of *S*, *E*, *I*, *Q*, *R* for $\tau _{1}=4.6292>\tau _{10}$, $\tau _{2}=2.299$ along with time *t*

After calculation, we can obtain $\tau _{10}=7.8879$, $\tau _{20}=6.3851$, $\tau _{*0}=2.9393$. The corresponding values of *ω* are as follows: $\omega _{10}=0.0927$, $\omega _{20}=0.1157$, $\omega _{30}=0.2514$. We take different combinations of *τ* as control variable to carry out numerical simulations. We choose some parameters, which are smaller than *τ*, to carry out numerical simulation, and the results are shown in Figs. 1, 3, 5 and 7. According to these figures, we can see that when *τ* is smaller than the critical value, system (2) is locally asymptotically stable at $G^{*}(1.58, 0.7016, 0.1353, 0.4959, 3.793)$. Figures 2, 4, 6 and 8 show the results of numerical simulation when the values of *τ* exceed the critical value, and it is easy to see that when *τ* exceeds the critical value, system (2) becomes unstable and there appears a Hopf bifurcation at $G^{*}(1.58, 0.7016, 0.1353, 0.4959, 3.793)$.

## Conclusions

In this paper, based on the model formulated in [25], we consider the influence of supply chain transmission, hierarchical quarantine rate and time delay, and then we develop a novel Susceptible–Expose–Infected–Quarantined–Recovered (SEIQR) COVID-19 propagation model with two delays. In the new model, we analyze the existence of a virus-free equilibrium and a virus-existence equilibrium. After analysis, we find that system (2) has only a virus-existence equilibrium, and has no virus-free equilibrium. Afterwards, we take the time delay as a bifurcation parameter, and research the local stability and the existence of a Hopf bifurcation for the virus-existence equilibrium. Then we get the result when *τ* is smaller than the key value, system (2) reaches a local stable state eventually; otherwise, system (2) becomes unstable and there appears a Hopf bifurcation. The direction of the Hopf bifurcation and the stability of bifurcating periodic solutions also have been determined, and some numerical simulations are used to prove the validity of the theoretical results.

Compared with the model in [25], we consider the situation that people may be infected by items in transmission. Besides, we assume that exposed individuals and infected individuals have different quarantine rates, and this measure makes our new model practical. Time delays are applied during analyzing the new model, too. In the future, we will consider the nonlinear infection rate, which would lead to the increase of exposed individuals and infected individuals.

## Data Availability

The data used to support the findings of this study are available from the corresponding author upon request.
